# Intratumoral *Fusobacterium nucleatum* is associated with better cancer-specific survival in head and neck cancer patients

**DOI:** 10.1080/20002297.2025.2487644

**Published:** 2025-04-01

**Authors:** José Guilherme Datorre, Mariana Bisarro dos Reis, Bruna Pereira Sorroche, Gustavo Ramos Teixeira, Silveli Suzuki Hatano, Ana Carolina de Carvalho, Ricardo Ribeiro Gama, Lidia Maria Rebolho Batista Arantes, Rui Manuel Reis

**Affiliations:** a Molecular Oncology Research Center, Barretos Cancer Hospital, Barretos, Brazil; b Pathology Department, Barretos Cancer Hospital, Barretos, Brazil; c Barretos School of Health Sciences Dr. Paulo Prata ­(FACISB), Barretos, Brazil; d Head and Neck Surgery Department, Barretos Cancer Hospital, Barretos, Brazil; e Life and Health Sciences Research Institute (ICVS), School of Medicine, University of Minho, Braga, Portugal

**Keywords:** *Fusobacterium nucleatum*, head and neck cancer, droplet digital PCR, Brazil, Biomarker

## Abstract

**Background:**

The oral microbiome, particularly *Fusobacterium nucleatum (Fn)*, has been implicated in head and neck cancers (HNC), influencing local immunity and Human Papillomavirus (HPV) status. Here, we evaluated the presence of *Fn* and its association with HPV infection, *TERT* promoter (*TERTp*) mutations, and patient outcomes.

**Materials and Methods:**

We analyzed 94 formalin-fixed paraffin-embedded (FFPE) tumor tissues from HNC patients previously evaluated for *TERTp* mutations. *Fn* DNA was detected using droplet digital PCR (ddPCR), and HPV status was determined via p16 immunohistochemistry in pre-treatment samples. Associations between *Fn* presence, clinicopathological features, HPV, and TERTp mutation status were assessed.

**Results:**

Tumors primarily originated from the oropharynx (70.2%) and oral cavity (29.8%). Tobacco and alcohol use were reported in 87.2% and 79.8% of cases, respectively. *Fn* was present in 59.6% of cases, with higher prevalence in oropharyngeal (62.1%) than oral cavity (53.6%) tumors. No significant associations were found between *Fn* and clinicopathological features, TERTp, or HPV status. However, patients with *Fn* positivity showed significantly improved cancer-specific survival (61.5% vs. 39.1%, *p* = 0.013), similar to HPV-positive patients (72.7% vs. 42.7%, *p* = 0.014).

**Conclusion:**

The presence of *Fusobacterium nucleatum* in HNC correlates with longer survival, highlighting its potential as a prognostic marker.

## Introduction

Head and neck carcinoma (HNC) affects the oral cavity, oropharynx, nasopharynx, hypopharynx, larynx, nasal cavity, paranasal sinuses, and salivary glands, ranking as the seventh most diagnosed cancer worldwide [1]. The HNC is a complex multifactorial disease influenced by genetic and environmental factors [1]. In developing countries, risk factors, such as tobacco exposure and/or excessive alcohol consumption, are key etiological agents [1]. In contrast, in developed countries, there is an increased number of oropharyngeal cancers associated with Human Papillomavirus (HPV) infection, especially subtypes HPV-16 and, in some cases, HPV-18 [1,2]. The oral microbiome ecosystem is highly diverse, and it is essential for oral health [2]. Recent studies showed that the microbiome, including the *Fusobacterium nucleatum* (*Fn*), plays a role in the development and progression of HNC, probably by modulating the local immune response [3–5].

Biomarkers for prognosis in HNC hold substantial promise for refining patient management. Among these, a molecular biomarker evaluated in several types of tumors is telomerase reverse transcriptase (*TERT*) promoter mutation (*TERTp*). *TERTp* identification has gained substantial attention due to HNCs revealing augmented expression of *TERT* transcripts, which has been associated with poor treatment outcomes and an increased risk of disease progression [6–8]. Additionally, the well-documented influence of HPV status in oropharyngeal cancer prognosis emphasizes the significance of viral biomarkers in understanding and predicting disease progression and patient outcomes [9].

In the present study, we aimed to evaluate the presence of *Fn* in head and neck cancers associated with HPV and *TERTp* status and further explore its potential as a prognostic biomarker in these patients.

## Patients/material and methods

### Patient samples

This retrospective study included 94 pre-treatment formalin-fixed paraffin-embedded (FFPE) tumor tissues from HNC patients treated at the Department of Head and Neck Surgery, Barretos Cancer Hospital, Brazil, between 2006 and 2012. The inclusion criteria were previously untreated patients with a primary HNC sample available. All individuals enrolled signed a written consent to participate and the project was approved by the Institutional Review Board (approval number 1506/2017).

The clinicopathological characteristics of the patients, along with the follow-up data, were extracted from the hospital’s medical records. Follow-up duration was defined as the time interval between the date of diagnosis and the date of the last documented clinical contact, death, or censoring event, whichever occurred first.

### DNA isolation and TERT promoter mutational analysis

DNA was isolated from FFPE, as previously reported [8]. Hematoxylin and eosin-stained sections corresponding to the paraffin-embedded tissue blocks were reviewed by an expert pathologist to confirm the diagnosis and assess the cellular composition of the samples. Only regions containing at least 80% tumor cells were selected for DNA extraction. From each block, up to 8 sections were obtained, each with a thickness of 10 µm and a surface area of up to 250 mm^2^. DNA was extracted using the QIAamp DNA FFPE Tissue Kit (Qiagen, Germany), quantified using the NanoDrop 2000C (Thermo Scientific™), and stored at − 20°C until further analysis. A pyrosequencing assay was performed to investigate the hotspot *TERT* promoter mutations, C228T and C250T, as previously reported on a PyroMark Q96ID system using PyroMark Gold reagents (Qiagen) [8].

### Detection of *Fusobacterium nucleatum* DNA by droplet digital PCR (ddPCR)

The presence of *Fusobacterium nucleatum* (*Fn*) was evaluated on tumor DNA by ddPCR. Specific probe and primer sequences targeting a conserved region of the *Fn* genome were employed, as previously described [10]. The sequences used were as follows: Fn forward primer: 5′-CAACCATTACTTTAACTCTACCATGTTCA-3′; Fn reverse primer: 5′-GTTGACTTTACAGAAGGAGATTATGTAAAAATC-3′; Fn FAM probe: 5′-GTTGACTTTACAGAAGGAGATTA-3′ [11].

ddPCR reaction was prepared with 20 ng of DNA, 1× ddPCR Supermix for Probes no dUTP (BioRad, USA), 0.25 µM of each primer, and 0.125 µM of probe in a total volume of 20 µL. The droplet was generated by QX100™ Droplet Generator (BioRad, USA). The PCR reaction was performed by cycling condition of an initial heating at 95°C for 10 minutes, and 40 cycles of denaturation at 94°C for 30 seconds, annealing at 60°C for 60 seconds, and a final heating at 98°C for 10 minutes. A Q×100droplet reader (BioRad, USA) measured the PCR amplification plate, and fluorescence amplitude data were collected using QuantaSoft Software (BioRad, USA). All experiments included optimization, analytical assessment of *Fn* DNA detection by ddPCR, determination of efficacy, and assay sensitivity as described previously [10]. Results were expressed as copies/reaction of total DNA added to the reaction. Furthermore, the cases were classified as negative and positive, according to the assay’s Limit of Detection (LoD) (LoD = 2.7 copies of *Fn*).

### p16 immunohistochemistry

Protein expression of high-risk HPV biomarker was evaluated by p16 immunohistochemistry as previously described [12], and it was considered positive when there was strong and widespread nuclear and cytoplasmic staining, characterized by intense brown staining easily distinguishable from background under low magnification (10× or 20×), present in at least 70% of tumor cells within the specimen. The 70% threshold was selected based on established guidelines [13,14] and internal validation studies [12]. Cases with faint, patchy, or focal staining were considered negative.

### Statistical analysis

Statistical analyses were performed using IBM SPSS 21.0 for Windows. The Chi-square test, Fisher’s exact test, and multivariate (Linear regression model) were used to compare the presence/absence of *Fn* between HNC patients’ pathological and clinical features. Survival curves were calculated using the Kaplan-Meier method, and differences between groups were compared using the log-rank test using R version 4.3.1 and the software IDE (Integrated Development Environment).

## Results

### Clinicopathological characterization of HNC patients

**Figure 1.**
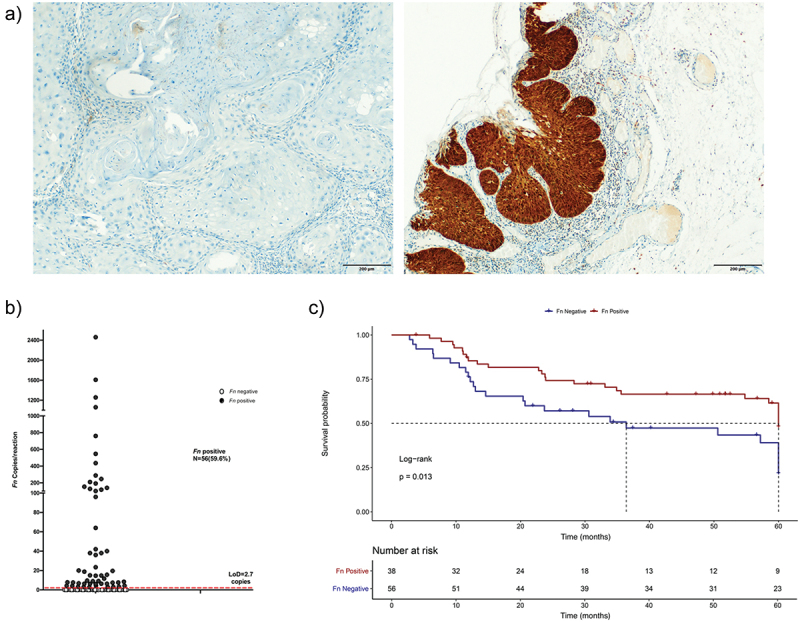
(a) Representative examples of negative (left) and positive (right) p16 immunostaining in head and neck cancer (HNC). (b) Absolute amount of *Fusobacterium nucleatum* (*Fn*) in HNC patients. A total of 94 samples were analyzed by ddPCR. Dot plots represent samples, and the dotted line represents the limit of detection (LoD) used to classify samples as negative (below the LoD) and positive (above the LoD). (c) Kaplan-Meier curve of HNC cancer-specific survival for patients based on the presence or absence of *Fn*.

The clinicopathological features of the 94 hNC cases are summarized in Table 1. The primary tumor sites included oral cavity (29.8%) and oropharynx (70.2%). Most of the patients were male (88.3%), with an average age of 58.47 years. Tobacco or alcohol consumption (current or former) was reported by 87.2% and 79.8%, respectively. Regarding the HPV status, 28.8% of oropharyngeal and 7.1% of oral cavity cancer patients were p16 positive (Figure 1a). Concerning *TERTp* mutations, we observed that 22.3% harbored hotspot mutations, namely 22.22% C228T and 4.76% C250T. The median follow-up of patients was 38.8 months (range 2.8 to 60 months).

**Table 1.** Demographic and clinicopathological features of the 94 head and neck carcinoma (HNC) cases.

Variables	N (%)	Oropharynx N = 66%)	Oral cavity N = 28%)
**Gender**			
Male	83 (88.3)	62 (93.9)	21 (75.0)
Female	11 (11.7)	4 (6.1)	7 (25.0)
**Age**			
≤60 years	60 (63.8)	43 (65.2)	17 (60.7)
>60 years	34 (36.2)	23 (34.8)	11 (39.3)
**Average age (standard deviation)**	58.5 (9.5)	58.0 (9.4)	59.5 (9.9)
**Tobacco consumption**			
No	12 (12.8)	9 (13.6)	3 (10.7)
Yes	82 (87.2)	57 (86.4)	25 (89.3)
**Alcohol consumption**			
No	17 (18.1)	9 (13.6)	8 (28.6)
Yes	75 (79.8)	55 (83.3)	20 (71.4)
Missing	2 (2.1)	2 (3.0)	0 (0.0)
**Tobacco and alcohol in combination**			
Yes	68 (72.3)	16 (24.2)	8 (28.6)
No	24 (25.5)	48 (72.7)	20 (71.4)
Missing	2 (2.1)	2 (3.0)	0 (0.0)
**Anatomical site**			
Oral cavity	28 (29.8)	0 (0.0)	28 (100.0)
Oropharynx	66 (70.2)	66 (100.0)	0 (0.0)
**T stage**			
T1	5 (5.4)	3 (4.5)	2 (7.4)
T2	28 (30.1)	17 (25.8)	11 (40.7)
T3	35 (37.6)	27 (40.9)	8 (29.6)
T4	25 (26.9)	19 (28.8)	6 (22.2)
**N stage**			
N0	24 (25.5)	11 (16.7)	13 (46.4)
N1	18 (19.1)	12 (18.2)	6 (21.4)
N2	42 (44.7)	36 (54.5)	6 (21.4)
N3	9 (9.6)	7 (10.6)	2 (7.1)
NX	1 (1.1)	0 (0.0)	1 (3.6)
**M stage**			
M0	94 (100.0)	66 (100.0)	28 (100.0)
M1	0 (0.0)	0 (0.0)	0 (0.0)
**Clinical TNM stage**			
I/II	15 (16.0)	7 (10.6)	8 (28.6)
III/IV	79 (84.0)	59 (89.4)	20 (71.4)
**HPV Status**			
Negative	73 (77.7)	47 (71.2)	26 (92.9)
Positive	21 (22.3)	19 (28.8)	2 (7.1)
***TERT*****p mutation**			
Mutant (C228T/C250T)	16 (17.0)	28 (42.4)	17 (60.7)
Wildtype	45 (47.9)	5 (7.6)	11 (39.3)
Missing	33 (35.1)	33 (50.0)	0 (0.0)
**Life status**			
Alive (without cancer)	32 (34.0)	26 (39.4)	6 (21.4)
Alive (with cancer)	5 (5.3)	5 (7.6)	0 (0.0)
Dead (by cancer)	50 (53.2)	34 (51.5)	16 (57.1)
Dead (other causes)	7 (7.4)	1 (1.5)	6 (21.4)

### Presence and association of intratumoral *Fusobacterium nucleatum* (Fn)

The presence of *Fn* in HNC was evaluated by ddPCR, and we observed positive *Fn* in 59,5% (59/94) of cases, with values ranging from 2.7 to 2,460 copies/reaction (Figure 1b).

Next, we evaluated the association of *Fn* presence with patients’ clinicopathological features (Table 2). In univariate and multivariate analyses, *Fn* was not associated with any clinical and clinicopathological characteristics (Table 2). Likewise, no association was found between *Fn* presence and HPV and *TERTp* mutation status in this cohort (Table 2).

**Table 2.** Association of *Fusobacterium nucleatum* with clinicopathological features.

	Univariate						Multivariate			
		*Fusobacterium nucleatum*								
Variable		Negative		Positive		*p* value		HR	95%CI	*p* value
		N	%	N	%					
Gender	Male	35	92.1	48	85.7	0.344				
	Female	3	7.9	8	14.3					
Age	≤60 years	22	57.9	38	67.9	0.324				
	>60 years	16	42.1	18	32.1					
Tobacco consumption	No	2	5.3	10	17.9	0.073	No	Ref	Ref	0.430
	Yes	36	94.7	46	82.1		Yes	0.462	0.068–3.153	
Alcohol consumption	No	4	10.8	13	23.6	0.12	No	Ref	Ref	0.246
	Yes	33	89.2	42	76.4		Yes	0.387	0.078–1.923	
Tobacco and alcohol consumption	No	6	16.2	18	32.7	0.077				
	Yes	31	83.8	37	67.3					
Anatomical site	Oral cavity	13	34.2	15	26.8	0.44				
	Oropharynx	25	65.8	41	73.2					
T stage	T1/2	17	45.9	18	32.1	0.179	T1/2	Ref	Ref	0.193
	T3/4	20	54.1	38	67.9		T3/4	2.492	0.631–9.846	
N stage	N0	13	34.2	11	19.6	0.112	N0	Ref	Ref	0.847
	*N* > 1	25	65.8	45	80.4		*N* > 1	1.152	0.275–4.816	
Clinical TNM stage	I/II	9	23.7	6	10.7	0.092	I/II	Ref	Ref	0.939
	III/IV	29	76.3	50	89.3		III/IV	1.087	0.127–9.290	
HPV Status	Negative	30	78.9	43	76.8	0.805				
	Positive	8	21.1	13	23.2					
*TERTp* mutation	WT	15	62.5	30	81.1	0.107	WT	Ref	Ref	0.127
	MT	9	37.5	7	18.9		MT	0.381	0.111–1.315	

HR: hazard ratio. CI: confidence interval.

We further explored the association of *Fn* with patient survival and found that the presence of *Fn* was associated with prolonged cancer-specific survival (hazard ratio = 0.507, 95% confidence interval: 0.290–0.885, *p* = 0.017), with a median survival time of 60.0 months in this group compared to 36.4 months in the *Fn*-absent group (log rank *p* = 0.013, Figure 1c).

## Discussion

Our study interrogated the intratumoral presence of *Fusobacterium nucleatum* (*Fn*) in a Brazilian head and neck cancer (HNC) population and associated with epidemiological, clinical, and molecular features.

*Fn* is a well-studied and common oral opportunistic bacterium, and several studies have suggested its role in periodontal disease [15,16]. Moreover, the involvement of *Fn* in periodontal disease and its potential role in cancer pathogenesis has been related to *Fn*-driven inflammation, which contributes to disease progression in a model of oral tumorigenesis [15,16].

Herein, using a very sensitive ddPCR assay, we found that approximately 60% of our cases showed the presence of *Fn* intratumorally. These results follow the literature studies that reported the abundant presence of *Fn*, with some studies reporting up to 80% of cases, and importantly, its presence was significantly higher in tumors than in normal counterpart tissues [4,17–23].

We further investigated whether *Fn* presence was associated with clinicopathological features and HPV and *TERTp* mutation status. We did not observe any significant association. Nevertheless, some studies reported associations of *Fn* with older and non-drinking patients [22]. *Fn* association with HPV is not consensual; some studies report to be mutually exclusive events, whereas others suggest that it depends on other features, such as smoking and alcohol consumption [17,24].

Importantly, we found that *Fn* presence was associated with a better patient outcome. These results align with Neuzillet and co-authors [22], who evaluated 212 hNCs by qRT-PCR. *Fn* was associated with a favorable prognosis, namely significantly longer overall, relapse-free, and metastasis-free survival [22]. Similarly, Chen and co-authors [25], analyzing the microbiome profile by metabarcoding of 68 hNC, observed that *Fn* presence was associated with lower tumor stage and improved cancer-specific survival. Moreover, Chan et al. [26], 166 cases, also reported a significant association of *Fn* with early T and N stages and better 3-year disease-specific survival. At variance, Desai et al. [24] showed that *Fn* is associated with inflammation and poor survival in HNC cancer. Aligned with these later findings, *in vitro* and *in vivo* studies showed the tumor-promoting effects of *Fn* in oral squamous cell carcinoma [27,28].

Our findings differ from two other studies by the same group using The Cancer Genome Atlas (TCGA) database: one reported that high *Fusobacterium* levels were linked to good prognosis in HNC only in the absence of alcohol and/or smoking [17], while the other found no association with survival [29]. These discrepancies could be related to the distinct study designs, methodological approaches, and patient cohorts. While these studies analyzed *Fusobacterium* at the genus level using whole-genome/exome sequencing on a heterogeneous HNC cohort, our study employed ddPCR to specifically quantify *Fn* at the species level in oropharyngeal and oral cavity cancers. Species-specific effects of *Fn* may have been diluted or overlooked in genus-level analyses, as *Fn* represents approximately 50% of the *Fusobacterium* species found in HNC [30]. Other study identified 62 *Fusobacterium* species, and revealed that these species exhibit lineage-specific associations with distinct features of colorectal cancer [31]. Additionally, anatomic sites and their distinct etiological features and microenvironments may play a role, as the microbiome and its interactions in the oral cavity and oropharynx differ from other head and neck regions [32], where *Fn* may exert beneficial effects that promote immune responses or enhance therapeutic efficacy rather than solely contributing to tumor progression.

In colorectal cancer, *Fn* is often associated with tumor progression and poor prognosis due to its ability to promote inflammation, immune evasion, and chemoresistance [33]. However, in oropharyngeal and oral cavity cancers, the tumor microenvironment and immune response may differ significantly. For example, *Fn* might induce a localized immune response that could paradoxically enhance anti-tumor immunity in certain contexts, leading to improved survival [22].

Further potential explanations include geographical and ethnic variations [32,34], which influence microbiome composition and host immune interactions, as well as differences in oral health status, such as periodontal disease or oral hygiene practices, which can modulate the abundance and activity of *Fn* and its interaction with other species in the tumor microenvironment [35,36]. These factors, combined with the influence of lifestyle factors like smoking and alcohol, may explain the divergent findings. It is important to note that this study did not collect specific data for all of these variables for the included patients. As a result, we are unable to assess the extent to which these factors may have contributed to the observed associations. Further studies are needed to validate these findings and clarify the underlying mechanisms involving *Fn* before any clinical applications can be considered.

## Conclusion

Our study suggests that the presence of *Fn* was associated with an improved cancer-specific survival rate, akin to the favorable prognosis seen in HPV-positive cases, highlighting its potential prognostic relevance in HNC.

## Key messages

- The presence of *Fusobacterium nucleatum (Fn)* is linked to significantly improved cancer-specific survival in head and neck cancer (HNC) patients.
- *Fn’s* impact on survival is independent of HPV positivity and TERT promoter mutation status.
- These findings suggest that *Fn* may constitute a potential biomarker for risk stratification in HNC.

## Data Availability

Data is available upon request.

## References

[1] Gormley M, Creaney G, Schache A, et al. Reviewing the epidemiology of head and neck cancer: definitions, trends and risk factors. Br Dent J. 2022;233(9):780–7. doi: 10.1038/s41415-022-5166-x 36369568 PMC9652141

[2] Kaan AM, Brandt BW, Buijs MJ, et al. Comparability of microbiota of swabbed and spit saliva. Eur J Oral Sci. 2022;130(2):e12858. doi: 10.1111/eos.12858 35218587 PMC9305955

[3] Desai S. Influence of pathogens on host genome and epigenome in development of head and neck cancer. Cancer Rep. 2023;6(11):e1846. doi: 10.1002/cnr2.1846 PMC10644332 37322598

[4] Sahin TK, Sonmezer MC. The role of the microbiome in head and neck squamous cell cancers. Eur Arch Otorhinolaryngol. 2025;282(2):623–637. doi: 10.1007/s00405-024-08966-6 39306588

[5] Goyal N, Day A, Epstein J, et al. Head and neck cancer survivorship consensus statement from the American head and neck Society. Laryngoscope Investig Otolaryngol. 2022;7(1):70–92. doi: 10.1002/lio2.702 PMC8823162 35155786

[6] Yu Y, Fan D, Song X, et al. TERT promoter mutations are enriched in oral cavity cancers and associated with locoregional recurrence. JCO Precis Oncol. 2021;5(5):1259–1269. doi: 10.1200/PO.20.00515 PMC8345918 34381934

[7] Boscolo-Rizzo P, Tirelli G, Polesel J, et al. TERT promoter mutations in head and neck squamous cell carcinoma: a systematic review and meta-analysis on prevalence and prognostic significance. Oral Oncol. 2023;140:106398. doi: 10.1016/j.oraloncology.2023.106398 37075587

[8] Arantes L, Cruvinel-Carloni A, de Carvalho AC, et al. TERT promoter mutation C228T increases risk for tumor recurrence and death in head and neck cancer patients. Front Oncol. 2020;10:1275. doi: 10.3389/fonc.2020.01275 32850388 PMC7399085

[9] Ang KK, Harris J, Wheeler R, et al. Human papillomavirus and survival of patients with oropharyngeal cancer. N Engl J Med. 2010;363(1):24–35. doi: 10.1056/NEJMoa0912217 20530316 PMC2943767

[10] Datorre JG, de Carvalho AC, Dos Reis MB, et al. Accuracy and clinical relevance of intra-tumoral *fusobacterium nucleatum* detection in formalin-fixed paraffin-embedded (FFPE) tissue by droplet digital PCR (ddPCR) in colorectal cancer. Diagnostics (Basel). 2022;12(1):12. doi: 10.3390/diagnostics12010114 PMC8775036 35054281

[11] Mima K, Sukawa Y, Nishihara R, et al. *Fusobacterium nucleatum* and T cells in colorectal carcinoma. JAMA Oncol. 2015;1(5):653–661. doi: 10.1001/jamaoncol.2015.1377 26181352 PMC4537376

[12] Santos Carvalho R, Scapulatempo-Neto C, Curado MP, et al. HPV-Induced oropharyngeal squamous cell carcinomas in Brazil: prevalence, trend, clinical, and epidemiologic characterization. Cancer Epidemiol Biomarkers Prev. 2021;30:1697–1707. 34155066 10.1158/1055-9965.EPI-21-0016

[13] Singhi AD, Westra WH. Comparison of human papillomavirus in situ hybridization and p16 immunohistochemistry in the detection of human papillomavirus-associated head and neck cancer based on a prospective clinical experience. Cancer. 2010;116(9):2166–2173. doi: 10.1002/cncr.25033 20186832

[14] Begum S, Gillison ML, Ansari-Lari MA, et al. Detection of human papillomavirus in cervical lymph nodes: a highly effective strategy for localizing site of tumor origin. Clin Cancer Res. 2003;9(17):6469–6475. 14695150

[15] Chen Y, Huang Z, Tang Z, et al. More than just a periodontal pathogen -the research progress on *fusobacterium nucleatum*. Front Cell Infect Microbiol. 2022;12:815318. 35186795 10.3389/fcimb.2022.815318 PMC8851061

[16] Fernandes BB, Datorre JG, Vazquez FDL, et al. Association of *fusobacterium nucleatum* levels by ddPCR in oral rinse samples with periodontal disease in oral squamous cell carcinoma patients and in controls. J Oral Pathol Med. 2024;53(10):657–666. doi: 10.1111/jop.13580 39444169

[17] Hamada M, Nishiyama K, Nomura R, et al. Clinical relationships between the intratumoral microbiome and risk factors for head and neck cancer. Heliyon. 2024;10(20):e39284. doi: 10.1016/j.heliyon.2024.e39284 39497974 PMC11533578

[18] Shin JM, Luo T, Kamarajan P, et al. Microbial communities associated with primary and metastatic head and neck squamous cell carcinoma – a high fusobacterial and low streptococcal signature. Sci Rep. 2017;7(1):9934. doi: 10.1038/s41598-017-09786-x 28855542 PMC5577109

[19] Zhao H, Chu M, Huang Z, et al. Variations in oral microbiota associated with oral cancer. Sci Rep. 2017;7(1):11773. doi: 10.1038/s41598-017-11779-9 28924229 PMC5603520

[20] Liu Y, Li Z, Qi Y, et al. Metagenomic analysis reveals a changing microbiome associated with the depth of invasion of oral squamous cell carcinoma. Front Microbiol. 2022;13:795777. doi: 10.3389/fmicb.2022.795777 35222330 PMC8863607

[21] Pratap Singh R, Kumari N, Gupta S, et al. Intratumoral microbiota changes with tumor stage and influences the immune signature of oral squamous cell carcinoma. Microbiol Spectr. 2023;11(4):e0459622. doi: 10.1128/spectrum.04596-22 37409975 PMC10434029

[22] Neuzillet C, Marchais M, Vacher S, et al. Prognostic value of intratumoral *fusobacterium nucleatum* and association with immune-related gene expression in oral squamous cell carcinoma patients. Sci Rep. 2021;11(1):7870. doi: 10.1038/s41598-021-86816-9 33846399 PMC8041800

[23] Torralba MG, Aleti G, Li W, et al. Oral microbial species and virulence factors associated with oral squamous cell carcinoma. Microb Ecol. 2021;82(4):1030–1046. doi: 10.1007/s00248-020-01596-5 33155101 PMC8551143

[24] Desai S, Dharavath B, Manavalan S, et al. *Fusobacterium nucleatum* is associated with inflammation and poor survival in early-stage hpv-negative tongue cancer. NAR Cancer. 2022;4:zcac006. 35252868 10.1093/narcan/zcac006 PMC8894079

[25] Chen Z, Wong PY, Ng CWK, et al. The intersection between oral microbiota, host gene methylation and patient outcomes in head and neck squamous cell carcinoma. Cancers (Basel). 2020;12(11):12. doi: 10.3390/cancers12113425 PMC7698865 33218162

[26] Chan JYK, Cheung MK, Lan L, et al. Characterization of oral microbiota in HPV and non-hpv head and neck squamous cell carcinoma and its association with patient outcomes. Oral Oncol. 2022;135:106245. doi: 10.1016/j.oraloncology.2022.106245 36375420

[27] Nie F, Zhang J, Tian H, et al. The role of CXCL2-mediated crosstalk between tumor cells and macrophages in *Fusobacterium nucleatum*-promoted oral squamous cell carcinoma progression. Cell Death Dis. 2024;15(4):277. doi: 10.1038/s41419-024-06640-7 38637499 PMC11026399

[28] Li Z, Liu Y, Huang X, et al. F. Nucleatum enhances oral squamous cell carcinoma proliferation via E-cadherin/β-catenin pathway. BMC Oral Health. 2024;24(1):518. doi: 10.1186/s12903-024-04252-3 38698370 PMC11064238

[29] Hamada M, Inaba H, Nishiyama K, et al. Potential role of the intratumoral microbiota in prognosis of head and neck cancer. IJMS. 2023;24(20):24. doi: 10.3390/ijms242015456 PMC10607002 37895136

[30] Bronzato JD, Bomfim RA, Edwards DH, et al. Detection of fusobacterium in oral and head and neck cancer samples: a systematic review and meta-analysis. Arch Oral Biol. 2020;112:104669. doi: 10.1016/j.archoralbio.2020.104669 32028171

[31] Bi D, Zhu Y, Gao Y, et al. Profiling fusobacterium infection at high taxonomic resolution reveals lineage-specific correlations in colorectal cancer. Nat Commun. 2022;13(1):3336. doi: 10.1038/s41467-022-30957-6 35680952 PMC9184491

[32] Yalamarty R, Magesh S, John D, et al. The intratumor microbiome varies by geographical location and anatomical site in head and neck squamous cell carcinoma. Curr Probl Cancer. 2024;50:101100. 38820649 10.1016/j.currproblcancer.2024.101100

[33] Galasso L, Termite F, Mignini I, et al. Unraveling the role of *fusobacterium nucleatum* in colorectal cancer: molecular mechanisms and pathogenic insights. Cancers (Basel). 2025;17(3):368. 39941737 10.3390/cancers17030368 PMC11816155

[34] Mason MR, Nagaraja HN, Camerlengo T, et al. Deep sequencing identifies ethnicity-specific bacterial signatures in the oral microbiome. PLOS ONE. 2013;8:e77287. 24194878 10.1371/journal.pone.0077287 PMC3806732

[35] Sulaiman Y, Pacauskiene IM, Sadzeviciene R, et al. Oral and gut microbiota Dysbiosis due to periodontitis: systemic implications and links to gastrointestinal cancer: a narrative review. Medicina (Kaunas). 2024;60(9):60. doi: 10.3390/medicina60091416 PMC11433653 39336457

[36] Brennan CA, Garrett WS. *Fusobacterium nucleatum* — symbiont, opportunist and oncobacterium. Nat Rev Microbiol. 2019;17(3):156–166. doi: 10.1038/s41579-018-0129-6 30546113 PMC6589823

